# Midlife blood pressure predicts future diastolic dysfunction independently of blood pressure

**DOI:** 10.1136/heartjnl-2015-308836

**Published:** 2016-04-07

**Authors:** Arjun Kumar Ghosh, Alun David Hughes, Darrel Francis, Nishi Chaturvedi, Denis Pellerin, John Deanfield, Diana Kuh, Jamil Mayet, Rebecca Hardy

**Affiliations:** 1 International Centre for Circulatory Health, National Heart and Lung Institute, Imperial College, London, UK; 2 Barts Heart Centre, St Bartholomew's Hospital, Barts Health NHS Trust, London, UK; 3 Institute of Cardiovascular Science, University College London, London, UK; 4 Medical Research Council Unit for Lifelong Health and Ageing, University College London, London, UK

## Abstract

**Objectives:**

High blood pressure (BP) is associated with diastolic dysfunction, but the consequence of elevated BP over the adult life course on diastolic function is unknown. We hypothesised that high BP in earlier adulthood would be associated with impaired diastolic function independent of current BP.

**Methods:**

Participants in the Medical Research Council National Survey of Health and Development birth cohort (n=1653) underwent investigations including echocardiography at age 60–64 years. The relationships between adult BP, antihypertensive treatment (HTT) and echocardiographic measures of diastolic function were assessed using adjusted regression models.

**Results:**

Increased systolic BP (SBP) at ages 36, 43 and 53 years was predictive of increased E/e′ and increased left atrial volume. These effects were only partially explained by SBP at 60–64 years and increased left ventricular mass. HTT was also associated with poorer diastolic function after adjustment for SBP at 60–64 years. Faster rates of increase in SBP in midlife were also associated with increased poorer diastolic function.

**Conclusions:**

High SBP in midlife is associated with poorer diastolic function at age 60–64 years. Early identification of individuals with high BP or rapid rises in BP may be important for prevention of impaired cardiac function in later life.

## Introduction

With an ageing population, and better postmyocardial infarction survival, the burden of congestive heart failure is increasing and represents a major public health challenge.^1^ Diastolic dysfunction is common in people over 45 years with a prevalence of 28% in a large community study,^1^ and is a precursor of heart failure with preserved ejection fraction (HFPEF).^2^ Echocardiographic measures of diastolic dysfunction and elevated left ventricular (LV) filling pressure also predict a threefold increased cardiac mortality and a twofold increased all-cause mortality in those with normal ejection fractions.^2^ ^3^ High systolic blood pressure (SBP) is an important cause of diastolic dysfunction, and there is evidence that raised antecedent SBP in early and midadulthood is associated with increased risk of heart failure,^4^ and elevated risk of cardiovascular mortality independent of current blood pressure (BP).^5^ However, there has been limited work investigating the effects of longitudinal changes in BP over adulthood on diastolic function.^6^ We have previously shown that increased SBP in adult midlife is associated with increased LV mass in later life independent of later SBP and that rapid rises in midlife SBP might play a key role.^7^ We hypothesised that a similar relationships may exist between SBP and diastolic function and also aimed to determine the extent to which this might be due to increased LV mass. In addition, we examined the association between antihypertensive treatment (HTT) and subsequent diastolic function.

## Methods

### Study patients

The UK Medical Research Council National Survey of Health and Development (MRC NSHD) is a prospective birth cohort study of singleton births that occurred in 1 week of March 1946 in England, Scotland and Wales (5362 births; 2547 women, 2815 men). Follow-up has included over 20 contacts with the whole cohort between birth and the most recent data collection when the participants were between 60 and 64 years of age.^7^

Study members still alive and with a known current address in England, Scotland or Wales were invited for an assessment at one of six clinical research facilities (CRFs) or to be visited by a research nurse at home. Invitations were not sent to those who had died (N=778), who were living abroad (N=570), had previously withdrawn from the study (N=594) or had been lost to follow-up (N=564). Of the 2856 invited participants, 2229 (78%) were assessed: 1690 (59%) attended a CRF and the remaining 539 were visited at home. Echocardiography was only carried out at the CRF (N=1653). The participating sample remains broadly representative of native born British men and women of the same age.^8^

Ethical approval was obtained from the Central Manchester Research Ethics Committee (07/H1008/168) and the Scotland A Research Ethics Committee. Written informed consent was obtained from each study member at each stage of data collection.

### Anthropometry and BP measurement

Height and weight were measured at the clinic visit and body mass index (BMI) calculated. Sitting brachial BP was measured in the upper right arm with an appropriately sized cuff after 5 min of rest at 53 years and 60–64 years with the second measurement used in analyses, or the first measure where the second was missing. A Hawksley random zero sphygmomanometer (Hawksley & Sons, Lancin, UK; a state of the art machine at the time) was used at 36 and 43 years.^5^ ^9^ A validated oscillometric device (Omron HEM-705) was used in later in home visits. To enable comparison of BP measured by the different machines, the measurements from the random zero sphygmomanometer were adjusted using published conversion equations.^10^

### Antihypertensive treatment and diabetes status

Prior to clinic attendance, study members completed a postal questionnaire that included details of current medication. Antihypertensive medications for the last two rounds were classified according to International Classification of Diseases and related Health Problems classification.^11^ Self-reported and doctor diagnosed type 2 diabetes mellitus (T2DM) was obtained from the postal questionnaires and reports at earlier follow-ups.

### Echocardiography

Of the 1690 participants who attended a clinic, 1653 (798 men and 855 women, mean age 63.3±1.1 years (1SD)) underwent echocardiography using GE Vivid I machines (GE, Connecticut, USA) and 1576 had at least one analysable image (95%). Echocardiographic images were obtained from parasternal long axis and short axis, apical five-chamber, four-chamber, three-chamber, two-chamber and aortic views along with conventional and tissue Doppler in the four-chamber view. Image analysis was carried out by three experienced British Society of Echocardiography-accredited readers including the author (AKG) masked to patient identity using GE EchoPac software.

The following markers of diastolic function were measured in accordance with American Society of Echocardiography/European Association of Echocardiography recommendations—ratio of early (E) to late (A) transmitral Doppler flow (E/A), early (e′) myocardial velocity at the mitral valve annulus (average of septal and lateral wall measures) e′, E/e′ and ratio of early and late (a′) myocardial velocities at the mitral valve annulus (average of septal and lateral wall measures) e′/a′.^12^ E/e′ was calculated as an estimate of LV filling pressure.^12^ Left atrial volume indexed to body surface area (LAVI) was also examined as a marker of chronically elevated LV filling pressures.^13^

Quality control measures included standardised training for senior, experienced echocardiographers and readers, echocardiographer observation by trained echocardiographers, periodic reader and echocardiographer review and refresher sessions, phantom studies on ultrasound machines and continuous quality control audit throughout the period of data collection. Blind duplicate reading reproducibility studies (n=70 on two occasions) were carried out to establish inter-reader and intrareader variability. These showed excellent reproducibility (intraclass correlation coefficients were >0.90 for most measurements).

### Statistical analysis

Statistical analysis was performed using Stata V.14.1 (StataCorp LP, USA). Initially, separate regression models investigated the association between SBP at each of the 4 ages at which it was measured and measures of diastolic function at 60–64 years, with adjustment for sex, age at clinic visit and clinic attended. We examined whether associations were linear by inspection of residuals. Given evidence that cardiac function may differ by sex,^1^ we also investigated whether associations with SBP were subject to effect modification by sex through inclusion of a sex×SBP term in models, but this was not statistically significant in any model. Consequently, we show data from models including both sexes that were adjusted for sex. HTT at the same age as BP measurement was then added to the models. Additional models assessed whether earlier SBP remained predictive once current SBP was also included, that is, to what extent antecedent SBP was independent of current SBP. This is an important consideration given the anticipated correlations between SBP at different times within an individual (tracking). Further statistical models were constructed by including potential confounders BMI, T2DM, smoking and physical activity status at age 60–64 years. In a further model left ventricular mass indexed to body surface area (LVMI) was included with possible confounders to explore the extent to which LVMI might mediate the associations observed.

In order to maintain the sample size and minimise bias introduced by missing data in fully adjusted analyses, we employed a multiple imputation procedure to impute missing covariates. For each outcome, a total of 20 imputed datasets were obtained using chained equations implemented using imputation by chained equations in Stata. For the imputation models we included all variables in the final adjusted analytic model, each model, as well as the outcome and additional variables that helped predict the missing covariates. The regression coefficients and standard errors were calculated for each imputed dataset, and then combined using Rubin's rule.

To investigate whether rate of change in SBP at a particular period of midlife was more strongly associated with diastolic function, we calculated the change in SBP for the periods 36–43 years, 43–53 years and 53–60/64 years *conditional* on earlier SBP by modelling each SBP measure (from age 43 years) on the earlier measure(s) for each sex and saving the residuals. These residuals reflect SBP velocity and can be interpreted as the change in SBP in an individual above or below that expected on average in the sample given their earlier SBP.^14^ The residuals were standardised (mean=0 and SD=1) to allow a comparison of the relative strength of associations between periods. We subsequently fitted regression models including all these standardised changes with each of the measures of diastolic function as the outcome. This analysis was performed only for individuals who had all variables observed (complete case analysis). Two models were constructed: model 1—adjusted for age, sex and CRF attended; model 2—model 1+T2DM+BMI+smoking status+physical activity status+current HTT. p Values were calculated using Wald tests. Similar analyses were repeated for diastolic BP (DBP), pulse pressure (PP) and mean arterial pressure (MAP).

Sensitivity analyses were carried out to assess whether the associations with SBP remained unchanged if those who were hypertensive (SBP≥140 mm Hg or DBP≥90 mm Hg) were excluded.

## Results

Characteristics of participants with any echocardiography data at age 60–64 years are shown in table 1. Those with unanalysable echocardiograms had higher BMIs and heart rates, but BP did not differ.^7^ Additional participant characteristics are presented in online supplementary table S1.

10.1136/heartjnl-2015-308836.supp1 Supplementary table Cardiac risk factor characteristics of study participants

**Table 1:** Echocardiographic and cardiac risk factor characteristics of study participants

	All		Men		Women	
Variable (at age 60–64 years unless stated otherwise)	n	Result	n	Result	n	Result
Age, years	1626	63.2 (1.1)	786	63.2 (1.2)	840	63.3 (1.1)
BMI, kg/m^2^	1633	27.7 (4.6)	791	27.7 (4.0)	842	27.6 (5.2)
SBP, mm Hg	1633	135.7 (18.0)	791	139.0 (17.8)	842	132.7 (17.6)
SBP in those on HTT, mm Hg	347	137.4 (17.4)	179	138.2 (16.2)	168	131.8 (16.9)
SBP in those not on HTT, mm Hg	1120	134.9 (18.0)	518	136.1 (17.6)	602	129.9 (15.8)
SBP in those with unknown HTT status, mm Hg	166	137.4 (18.6)	92	138.1 (17.3)	74	132.1 (17.2)
SBP at age 53 years, mm Hg	1537	134.3 (19.1)	738	138.0 (18.9)	799	130.8 (18.5)
SBP at age 43 years, mm Hg	1522	123.7 (14.1)	735	128.1 (13.1)	787	119.6 (13.6)
SBP at age 36 years, mm Hg	1479	120.0 (13.7)	714	125.8 (12.8)	765	114.6 (12.3)
DBP, mm Hg	1633	77.3 (9.7)	791	79.0 (9.8)	842	75.7 (9.3)
DBP at age 53 years, mm Hg	1537	83.5 (11.9)	738	86.4 (11.9)	799	80.9 (11.2)
DBP at age 43 years, mm Hg	1522	80.5 (9.5)	735	83.4 (9.0)	787	77.8 (9.1)
DBP at age 36 years, mm Hg	1477	78.3 (9.6)	713	80.8 (9.4)	764	75.9 (9.3)
Heart rate, bpm	1630	68.9 (11.2)	789	67.4 (11.3)	841	70.3 (10.9)
IVSD, cm	1473	1.1 (0.2)	701	1.1 (0.2)	772	1.0 (0.2)
PWT, cm	1471	1.0 (0.2)	699	1.1 (0.2)	772	0.9 (0.2)
RWT	1469	0.4 (0.1)	699	0.4 (0.1)	770	0.4 (0.1)
LVM, g	1471	181.3 (59.3)	699	209.3 (60.3)	772	156.0 (45.4)
LVMI, g/m^2^	1471	95.7 (26.6)	699	104.1 (27.9)	772	88.1 (22.8)
Ejection fraction (%)	1459	68.7 (9.7)	692	67.2 (10.1)	767	69.9 (9.2)
E/A	1576	1.0 (0.3)	755	1.0 (0.3)	821	1.0 (0.3)
e′, cm/s	1533	8.8 (1.9)	727	8.9 (1.9)	806	8.8 (1.9)
E/e′	1490	7.9 (2.1)	701	7.5 (2.0)	789	8.3 (2.1)
e′/a′	1507	0.8 (0.2)	709	0.8 (0.2)	798	0.9 (0.2)
LAVI, mL/m^2^	1417	21.1 (7.2)	701	22.0 (7.4)	716	20.2 (7.0)

Data are mean (SD), median (interquartile range) or n (%) as appropriate.

BMI, body mass index; DBP, diastolic blood pressure; IVSD, interventricular septal thickness in diastole; LAVI, left atrial volume indexed to body surface area; LVM, left ventricular mass; LVMI, left ventricular mass indexed to body surface area; PWT, left ventricular posterior wall thickness in diastole; RWT, relative wall thickness; SBP, systolic blood pressure.

SBP from age 53 years was negatively associated with e′ (table 2). This relationship weakened with progressive risk factor adjustment. HTT was negatively associated with e′ in the minimally adjusted model from age 53 years.

**Table 2:** Regression between e′ at 60–64 years and SBP and antihypertensive treatment at four time points with further adjustment for covariables

		Model 1		Model 2		Model 3		Model 4		Model 5	
Independent variable		β (95% CI)	p Value	β (95% CI)	p Value	β (95% CI)	p Value	β (95% CI)	p Value	β (95% CI)	p Value
Age 36 (n=1533)	SBP	−0.006 (−0.014 to 0.002)	0.125	−0.006 (−0.014 to 0.002)	0.122	−0.001 (−0.009 to 0.007)	0.855	−0.002 (−0.010 to 0.006)	0.619	0.000 (−0.008 to 0.008)	0.931
Age 43 (n=1533)	SBP	−0.006 (−0.013 to 0.002)	0.128	−0.005 (−0.012 to 0.002)	0.171	0.002 (−0.067 to 0.010)	0.598	0.001 (−0.007 to 0.008)	0.816	0.000 (−0.009 to 0.008)	0.959
Age 53 (n=1533)	SBP	−0.011 (−0.017 to −0.006)	<0.001	−0.010 (−0.016 to −0.005)	<0.001	−0.005 (−0.011 to 0.001)	0.084	−0.004 (−0.010 to 0.002)	0.157	−0.002 (−0.006 to 0.009)	0.694
Age 60–64 (n=1533)	SBP	−0.017 (−0.022 to −0.012)	<0.001	−0.017 (−0.022 to −0.011)	<0.001	NR	NR	−0.015 (−0.020 to −0.009)	<0.001	−0.014 (−0.019 to −0.008)	<0.001
Age 36	HTT	NR	NR	0.112 (−0.846 to 1.070)	0.819	0.018 (−0.937 to 0.973)	0.971	0.039 (−0.910 to 0.989)	0.935	−0.110 (−1.157 to 0.937)	0.837
Age 43	HTT	NR	NR	−0.619 (−1.296 to 0.058)	0.073	−0.598 (−1.271 to 0.075)	0.081	−0.495 (−1.163 to 0.173)	0.146	−0.403 (−1.064 to 0.257)	0.231
Age 53	HTT	NR	NR	−0.333 (−0.635 to− 0.032)	0.030	−0.331 (−0.630 to −0.032)	0.030	−0.296 (−0.598 to 0.006)	0.054	−0.230 (−0.533 to 0.073)	0.136
Age 60–64	HTT	NR	NR	−0.253 (−0.490 to −0.016)	0.036	NR	NR	−0.162 (−0.407 to 0.082)	0.192	−0.094 (−0.340 to 0.152)	0.453

Imputed (n=1533 for all models—with valid outcome and SBP at age 60–64 years). The variable β is regression coefficient for e′ versus SBP (mm Hg) or antihypertensive treatment (HTT). Model 1: adjusted for age sex and CRF attended. Model 2: model 1+antihypertensive treatment (HTT) at given age (for SBP) or model 1+SBP at given age (for HTT). Model 3: model 2+SBP at 60–64 years. Model 4: model 3+T2DM+BMI+smoking status+physical activity status. Model 5: model 4+left ventricular mass indexed to body surface area. Numbers of individuals receiving HTT were 51 (2%), 107 (3%), 438 (15%) and 640 (27%) at age 36, 43, 53 and 60–64 years, respectively.

BMI, body mass index; CRF, clinical research facility; NR, not relevant; SBP, systolic blood pressure; T2DM, type 2 diabetes mellitus.

Broadly similar findings were observed when the associations between current and antecedent SBP and E/A were examined, although relationships tended to be weaker. SBP at 53 and 60–64 years was predictive of E/A and HTT at any age was unrelated to E/A (table 3). Data relating SBP measured at various ages to e′/a′ were consistent with observations for e′ and E/A (data not shown).

**Table 3:** Regression between E/A at 60–64 years and SBP and antihypertensive treatment at four time points with further adjustment for covariables

		Model 1		Model 2		Model 3		Model 4		Model 5	
Independent variable		β (95% CI)×10^−3^	p Value	β (95% CI)×10^−3^	p Value	β (95% CI)×10^−3^	p Value	β (95% CI)×10^−3^	p Value	β (95% CI)×10^−3^	p Value
Age 36 (n=1576)	SBP	0.3 (−0.8 to 1.5)	0.564	0.3 (−0.8 to 1.4)	0.598	0.9 (−0.2 to 2.1)	0.116	0.8 (−0.4 to 1.9)	0.190	0.8 (−0.3 to 2.0)	0.170
Age 43 (n=1576)	SBP	0.4 (−0.7 to 1.4)	0.481	0.4 (−0.6 to 1.5)	0.443	1.3 (0.2 to 2.3)	0.024	1.1 (0.06 to 2.2)	0.039	1.2 (0.1 to 2.2)	0.036
Age 53 (n=1576)	SBP	−1.4 (−2.2 to −0.7)	<0.001	−1.5 (−2.3 to −0.7)	<0.001	−1.0 (−1.8 to −0.1)	0.025	−0.7 (−1.5 to 0.1)	0.099	−0.7 (−1.5 to 0.2)	0.118
Age 60–64 (n=1576)	SBP	−1.8 (−2.6 to −1.1)	<0.001	−1.8 (−2.6 to −1.0)	<0.001	NR	NR	−1.5 (−2.2 to −0.7)	<0.001	−1.4 (−2.2 to −0.7)	<0.001
Age 36	HTT	NR	NR	70 (−80 to 220)	0.356	59 (−89 to 207)	0.430	70 (−73 to 214)	0.338	70 (−74 to 241)	0.339
Age 43	HTT	NR	NR	−35 (−131 to 62)	0.443	−36 (−133 to 61)	0.464	−27 (−131 to 77)	0.613	−17 (−127 to 93)	0.761
Age 53	HTT	NR	NR	27 (−16 to 70)	0.221	25 (−18 to 69)	0.248	40 (−3 to 83)	0.067	42 (−2 to 85)	0.059
Age 60–64	HTT	NR	NR	−25 (−58 to 9)	0.149	NR	NR	1.4 (−33 to 35)	0.936	2.9 (−32 to 37)	0.871

Imputed (n=1576 for all models—with valid outcome and SBP at age 60–64 years). The variable β is regression coefficient for E/A versus SBP (mm Hg) or antihypertensive treatment (HTT). Model 1: adjusted for age sex and CRF attended. Model 2: model 1+HTT at given age (for SBP) or model 1+SBP at given age (for HTT). Model 3: model 2+SBP at 60–64 years. Model 4: model 3+T2DM+BMI+smoking status+physical activity status. Model 5: model 4+left ventricular mass indexed to body surface area. Numbers of individuals receiving HTT were 51 (2%), 107 (3%), 438 (15%) and 640 (27%) at age 36, 43, 53 and 60–64 years, respectively.

BMI, body mass index; CRF, clinical research facility; NR, not relevant; SBP, systolic blood pressure; T2DM, type 2 diabetes mellitus.

SBP from 36 years onwards was positively associated with increased E/e′ (table 4). The relationship persisted after adjustment for age, sex, clinic attended, current HTT/SBP/BMI/T2DM/smoking/physical activity. SBP at age 53 years and HTT at age 53 years were also positively associated with LAVI (table 5). Analyses were repeated replacing SBP with DBP, PP or MAP should similar associations (data not shown).

**Table 4:** Regression between E/e′ at 60–64 years and SBP and antihypertensive treatment at four time points with further adjustment for covariables (n=1490 for all models—with valid outcome and SBP at age 60–64 years)

		Model 1		Model 2		Model 3		Model 4		Model 5	
Independent variable		β (95% CI)	p Value	β (95% CI)	p Value	β (95% CI)	p Value	β (95% CI)	p Value	β (95% CI)	p Value
Age 36 (n=1490)	SBP	0.015 (0.006 to 0.024)	0.001	0.015 (0.006 to 0.024)	0.001	0.007 (−0.002 to 0.016)	0.118	0.008 − (0.001 to 0.017)	0.072	0.006 (−0.003 to 0.015)	0.170
Age 43 (n=1490)	SBP	0.019 (0.011 to 0.027)	<0.001	0.017 (0.009 to 0.025)	<0.001	0.008 (0.000 to 0.017)	0.052	0.009 (0.001 to 0.017)	0.031	0.009 (0.000 to 0.017)	0.045
Age 53 (n=1490)	SBP	0.022 (0.017 to 0.028)	<0.001	0.020 (0.014 to 0.026)	<0.001	0.013 (0.007 to 0.019)	<0.001	0.011 (0.003 to 0.017)	0.001	0.010 (0.003 to 0.016)	0.003
Age 60–64 (n=1490)	SBP	0.024 (0.019 to 0.030)	<0.001	0.023 (0.018 to 0.029)	<0.001	NR	NR	0.021 (0.015 to 0.027)	<0.001	0.020 (0.014 to 0.025)	<0.001
Age 36	HTT	NR	NR	−0.06 (−1.18 to 1.06)	0.917	0.06 (−1.05 to 1.18)	0.909	0.004 (−1.08 to 1.09)	0.994	0.01 (−1.06 to 1.08)	0.981
Age 43	HTT	NR	NR	1.38 (0.62 to 2.13)	<0.001	1.34 (0.59 to 2.08)	<0.001	1.13 (0.39 to 1.87)	0.003	1.03 (0.30 to 1.75)	0.006
Age 53	HTT	NR	NR	0.72 (0.39 to 1.05)	<0.001	0.72 (0.39 to 1.04)	<0.001	0.62 (0.30 to 0.95)	<0.001	0.55 (0.23 to 0.88)	0.001
Age 60–64	HTT	NR	NR	0.69 (0.44 to 0.95)	<0.001	NR	NR	0.55 (0.29 to 0.82)	<0.001	0.48 (0.21 to 0.75)	<0.001

The variable β is regression coefficient for E/e′ versus SBP (mm Hg) or antihypertensive treatment (HTT). Model 1: adjusted for age sex and CRF attended. Model 2: model 1+HTT at given age (for SBP) or model 1+SBP at given age (for HTT). Model 3: model 2+SBP at 60–64 years. Model 4: model 3+T2DM+BMI+smoking status+physical activity status. Model 5: model 4+left ventricular mass indexed to body surface area. Numbers of individuals receiving HTT were 51 (2%), 107 (3%), 438 (15%) and 640 (27%) at age 36, 43, 53 and 60–64 years, respectively.

BMI, body mass index; CRF, clinical research facility; NR, not relevant; SBP, systolic blood pressure; T2DM, type 2 diabetes mellitus.

**Table 5:** Regression between LAVI at 60–64 years and SBP and antihypertensive treatment at four time points with further adjustment for covariables (n=1417 for all models—with valid outcome and SBP at age 60–64 years)

		Model 1		Model 2		Model 3		Model 4		Model 5	
Independent variable		β (95% CI)	p Value	β (95% CI)	p Value	β (95% CI)	p Value	β (95% CI)	p Value	β (95% CI)	p Value
Age 36 (n=1417)	SBP	0.042 (0.013 to 0.072)	0.005	0.041 (0.012 to 0.071)	0.006	0.035 (0.004 to 0.065)	0.025	0.38 (0.008 to 0.068)	0.014	0.024 (−0.005 to 0.053)	0.103
Age 43 (n=1417)	SBP	0.042 (0.013 to 0.070)	0.004	0.040 (0.011 to 0.069)	0.006	0.021 (0.001 to 0.061)	0.042	0.035 (0.005 to 0.064)	0.021	0.026 (−0.003 to 0.055)	0.079
Age 53 (n=1417)	SBP	0.056 (0.036 to 0.075)	<0.001	0.047 (0.027 to 0.068)	<0.001	0.043 (0.021 to 0.065)	<0.001	0.038 (0.016 to 0.060)	0.001	0.023 (0.023 to 0.045)	0.035
Age 60–64 (n=1417)	SBP	0.030 (0.009 to 0.051)	0.004	0.028 (0.008 to 0.050)	0.007	NR	NR	0.020 (−0.013 to 0.041)	0.066	−0.001 (−0.023 to 0.022)	0.944
Age 36	HTT	NR	NR	2.560 (−1.650 to 6.771)	0.232	2.769 (−1.433 to 6.971)	0.195	1.931 (−2.262 to 6.124)	0.365	2.022 (−1.935 to 5.979)	0.315
Age 43	HTT	NR	NR	2.179 (−0.393 to 4.751)	0.097	2.205 (−0.352 to 4.762)	0.091	1.388 (−1.204 to 3.981)	0.293	0.657 (−1.826 to 3.141)	0.603
Age 53	HTT	NR	NR	2.304 (1.139 to 3.469)	<0.001	2.326 (1.161 to 3.491)	<0.001	1.979 (0.799 to 3.159)	0.001	1.418 (0.260 to 2.576)	0.016
Age 60–64	HTT	NR	NR	1.290 (0.667 to 2.512)	0.001	–	NR	1.084 (0.134 to 2.033)	0.025	0.6453 (−0.466 to 1.371)	0.334

The variable β is regression coefficient for LAVI versus SBP (mm Hg) or antihypertensive treatment (HTT). Model 1: adjusted for age sex and CRF attended. Model 2: model 1+HTT at given age (for SBP) or model 1+SBP at given age (for HTT). Model 3: model 2+SBP at 60–64 years. Model 4: model 3+diabetes mellitus+body mass index+smoking status+physical activity status. Model 5: model 4+left ventricular mass indexed to body surface area. Numbers of individuals receiving HTT were 51 (2%), 107 (3%), 438 (15%) and 640 (27%) at age 36, 43, 53 and 60–64 years, respectively.

CRF, clinical research facility; LAVI, left atrial volume indexed to body surface area; NR, not relevant; SBP, systolic blood pressure.

From 43 years onwards, those on HTT had increased E/e′; this was not affected substantively by adjustment for current SBP; regression coefficients were slightly attenuated by adjustment for BMI/T2DM/smoking/physical activity although relationships remained statistically significant (table 4). We also investigated whether the effect of earlier SBP was mediated via LV hypertrophy by inclusion of LVMI into regression models. This was not the case.

We investigated whether relationships between elevated BP in earlier life and e′ and E/e′ might be driven by inclusion of people who went on to develop hypertension. However, when those who were hypertensive at 60–64 years (SBP ≥140 mm Hg or DBP ≥90 mm Hg) were excluded from the analysis, the associations remained very similar (data not shown).

**Figure 1:**
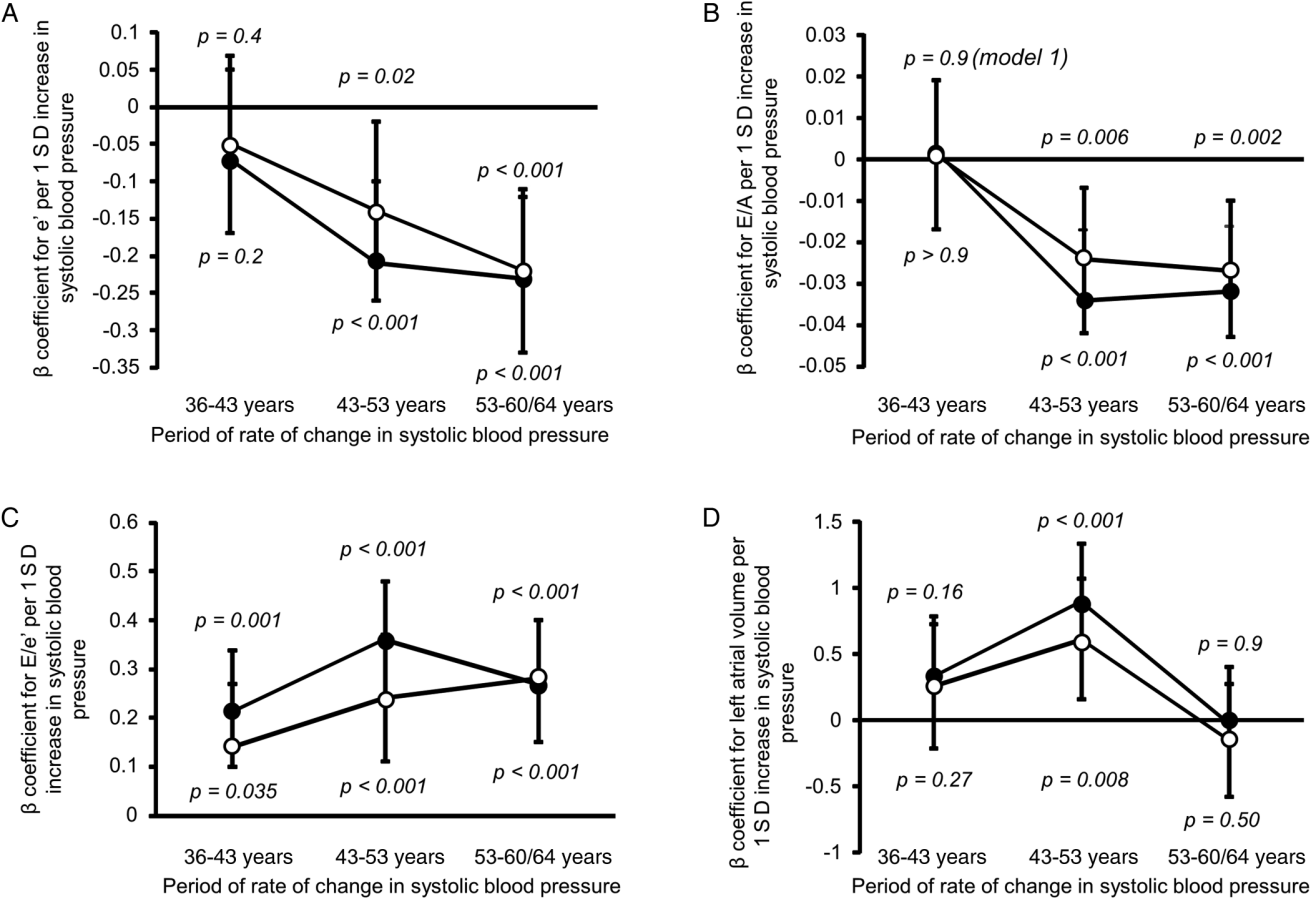
Association between standardised residuals of change in systolic blood pressure (SBP) over three time periods (36–43 years), (43–53 years) and (53–60 to 64 years) conditional on the earlier measure(s) and markers of diastolic function at age 60–64 years. The residuals can be interpreted as the standardised rate of change in SBP in an individual above or below that expected on average in the sample given their earlier SBP. (A) e′ at age 60–64 years (n=1172). (B) E/A at age 60–64 years (n=1190). (C) E/e′ at age 60–64 years (n=1120). (D) Left atrial volume indexed to body surface area at age 60–64 years (n=1054). Data points are β coefficients and the bars represent 95% CIs. Model 1 (•): adjusted for age, sex and clinical research facility attended. Model 2 (○): model 1+diabetes mellitus+body mass index+smoking status+physical activity status+current antihypertensive treatment. p Values were calculated using Wald tests and p<0.05 indicates a significant association between the rate of change in SBP in the specified period and the measure of diastolic function.

We looked to see whether there was a sensitive period when rate of change in SBP had most influence on diastolic function and filling pressure. Increased rates of rise in SBP over the age periods 43–53 and 53–60/64 years were also significantly associated with worse e′ (figure 1A) and E/A (figure 1B). Increased rate of rise in SBP at all age periods (36–43 years, 43–53 years and 53–60/64 years) was associated with increased E/e′ (figure 1C), although the relationship was strongest over the period 43–53 years in the minimally adjusted model. The rate of rise in the 43–53 years period was also associated with increased LAVI (figure 1D).

## Discussion

BP from early midlife predicted worse diastolic function and evidence of elevated filling pressure at age 60–64 years. This effect was independent of current BP, extended across the whole range of BP, and was not limited to people with hypertension or those who went on to develop hypertension at the time when diastolic function was assessed. We observed that those on HTT from early midlife had poorer diastolic function than those not on HTT even after current BP was accounted for. Further, we found that individuals showing comparatively rapid rises in SBP between 43–53 years and 53–60/64 years had worse subsequent diastolic function irrespective of their absolute level of SBP. Since the rise in BP in the UK population typically begins to accelerate around the fourth decade of life until it slows again in later life,^15^ we suggest that the period 40–60 years may represent a sensitive period when an accelerated rise in BP adversely influences future development of diastolic dysfunction.

There has been limited work in the past on relating antecedent BP with future diastolic function. Arnlöv *et al* reported that higher SBP and DBP at the age of 50 years were associated with decreased E/A measured 20 years later in the Swedish Uppsala cohort.^16^ Hypertension is thought to cause diastolic dysfunction via impaired LV relaxation and reduced LV compliance.^17^ Ultimately, the elevated filling pressures associated with severe diastolic dysfunction can lead to HFPEF^18^ or pulmonary oedema.^19^ Interestingly, Lee *et al* reported that higher BP and BMI in midlife were predictive of incident heart failure in later life in the Framingham study.^4^ Our data suggest that this relationship may be mediated at least in part via diastolic dysfunction.

Randomised clinical trials such as Antihypertensive and Lipid Lowering Treatment to Prevent Heart Attack Trial (ALLHAT) and HYpertension in the Very Elderly Trial (HYVET) have demonstrated the benefits of treating hypertension in terms of decreased incidence of new heart failure and heart failure admissions.^20^ However, optimal management of diastolic dysfunction remains unclear: trials in HFPEF have demonstrated little or no benefit with conventional heart failure medication.^21^ There is evidence for improvement in diastolic function with short-term HTT,^22^ ^23^ but longer-term HTT was not associated with improvement of diastolic function despite regression of LV mass in a substudy of the Anglo Scandinavian Cardiac Outcomes trial.^24^ Nevertheless, BP control is considered a cornerstone of management of these patients.^25^ In our cohort we found that from 43 years onwards, for any given level of BP (including for BP in the normal range, those on HTT had worse diastolic function at 60–64 years than those not on HTT). These data should not be interpreted as indicating that antihypertensive therapy has a negative effect on diastolic function, the observational design of the study precludes any such interpretation. Possible explanations of this finding include: inadequate long-term control of BP despite treatment^26^ ^27^—it should be noted that only approximately half of hypertensive participants were controlled to <140/90 mm Hg at age 60–64 years; or the accrual of some BP—related damage prior to initiation of treatment. If the latter were true, individuals at risk may need to be treated at an earlier age and/or to lower target BP.

Elevated antecedent BP is associated with LV hypertrophy,^7^ and this could contribute to impaired diastolic function; however, adjustment for LVMI only moderately attenuated associations between antecedent BP and diastolic function and it seems that associations, particularly for E/e′ and LAVI, are independent of increased LVMI. Some cross-sectional studies have found PP to be the best predictor of diastolic function,^28^ ^29^ but we found relatively similar associations between antecedent SBP, DBP, PP and MAP and diastolic function.

### Strengths and limitations

Our findings are derived from a nationally representative sample of native-born British people born in 1946.^8^ Using the life course approach in relating BP at different ages and change in BP over time to future diastolic function is unique and is a major strength of our study. This was only possible due to detailed repeated measurements of risk factors and recording of HTT in our study, which is the longest running birth cohort in the UK. Studies of birth cohorts have a number of advantages, notably that accounting for the effect of age is much less problematic. We used tissue Doppler as our primary measure of diastolic function, this has the advantage that it can distinguish normal diastolic function from pseudonormalisation.^30^ Pseudonormalisation will tend to attenuate relationships between antecedent BP and diastolic function assessed by E/A, and probably accounts for the generally weaker relationships seen using this measure of diastolic function. Missing data are inevitable in studies as long-running as the MRC NSHD (>60 years), although participant retention was good in comparison with other cohorts.^11^ Compared with those study members who attended clinic for clinical examination and echocardiography, those who only had clinical examinations at home visits had higher BMI and heart rates. However, participation of relatively more healthy individuals in the echocardiography study is, if anything, likely to have weakened relationships between BP and diastolic function. There were no measurements of BP prior to 36 years, and hence we cannot comment on the importance of BP in earlier life periods. We are limited to identifying a sensitive period from three intervals as the study has only four measures of BP. This lack of BP measurements in limits our ability to determine whether BP control was optimal between measurements. Echocardiography was only carried out in the last round of data collection; hence the possibility of diastolic dysfunction preceding the rise in BP cannot be excluded, although such a relationship seems unlikely.

## Conclusions

BP from the age of 36 years predicts diastolic function in people aged 60–64 years independently of current BP (for E/e′ and LAVI); faster increases in BP in midlife are particularly detrimental. People on HTT have more adverse diastolic function even when current BP is taken into account suggesting that early risk factor modification may be important to prevent the adverse effects of BP on diastolic function.

**Key messages**

**What is already known on this subject?**

- High blood pressure (BP) is associated with diastolic dysfunction in cross-sectional studies.
- Very little is known about the longitudinal effects of high BP and changes in BP over adult life on future diastolic function.

**What might this study add?**

- High blood pressure (BP) levels, and rises in BP in midadult life adversely affect diastolic function up to 28 years later.
- Hypertensive individuals have worse diastolic function even when their current BP was taken into account.

**How might this impact on clinical practice?**

- Early identification of those with fast rising blood pressure (BP) (even in the ‘normal’ BP range) may be important to prevent diastolic dysfunction in later life.

## References

[1] Redfield MM, Jacobsen SJ, Burnett JC, et al Burden of systolic and diastolic ventricular. Intern Med 2003;289:194–202. 10.1001/jama.289.2.194 12517230

[2] Halley CM, Houghtaling PL, Khalil MK, et al Mortality rate in patients with diastolic dysfunction and normal systolic function. Arch Intern Med 2011;171:1082–7. 10.1001/archinternmed.2011.244 21709107

[3] Bella JN, Palmieri V, Roman MJ, et al Mitral ratio of peak early to late diastolic filling velocity as a predictor of mortality in middle-aged and elderly adults: the Strong Heart Study. Circulation 2002;105:1928–33. http://www.ncbi.nlm.nih.gov/pubmed/11997279 (accessed 18 Aug 2014). 10.1161/01.CIR.0000015076.37047.D9 11997279

[4] Lee DS, Massaro JM, Wang TJ, et al Antecedent blood pressure, body mass index, and the risk of incident heart failure in later life. Hypertens 2007;50:869–76. 10.1161/HYPERTENSIONAHA.107.095380 17893376

[5] Allen N, Berry JD, Ning H, et al Impact of blood pressure and blood pressure change during middle age on the remaining lifetime risk for cardiovascular disease: the cardiovascular lifetime risk pooling project. Circulation 2012;125:37–44. 10.1161/CIRCULATIONAHA.110.002774 22184621 PMC3310202

[6] Arnlov J, Lind L, Zethelius B, et al Several factors associated with the insulin resistance syndrome are predictors of left ventricular systolic dysfunction in a male population after 20 years of follow-up. Am Heart J 2001;142:720–4. 10.1067/mhj.2001.116957 11579365

[7] Ghosh AK, Hardy RJ, Francis DP, et al Midlife blood pressure change and left ventricular mass and remodelling in older age in the 1946 British birth cohort study. Eur Hear J 2014;35:3287–95. 10.1093/eurheartj/ehu389 PMC4258225 25246483

[8] Stafford M, Black S, Shah I, et al Using a birth cohort to study ageing: representativeness and response rates in The National Survey of Health and Development. Eur J Ageing 2013;10:145–57. 10.1007/s10433-013-0258-8 23637643 PMC3637651

[9] Gray L, Lee I-M, Sesso HD, et al Blood pressure in early adulthood, hypertension in middle age, and future cardiovascular disease mortality: HAHS (Harvard Alumni Health Study). J Am Coll Cardiol 2011;58:2396–403. 10.1016/j.jacc.2011.07.045 22115646 PMC3253414

[10] Stang A, Moebus S, Möhlenkamp S, et al Algorithms for converting random-zero to automated oscillometric blood pressure values, and vice versa. Am J Epidemiol 2006;164:85–94. 10.1093/aje/kwj160 16675536

[11] Kuh D, Pierce M, Adams J, et al Cohort profile: updating the cohort profile for the MRC National Survey of Health and Development: a new clinic-based data collection for ageing research. Int J Epidemiol 2011;40:e1–9. 10.1093/ije/dyq231 21345808 PMC3043283

[12] Nagueh SF, Appleton CP, Gillebert TC, et al Recommendations for the evaluation of left ventricular diastolic function by echocardiography. J Am Soc Echocardiogr 2009;22:107–33. 10.1016/j.echo.2008.11.023 19187853

[13] Fatema K, Bailey KR, Petty GW, et al Increased left atrial volume index: potent biomarker for first-ever ischemic stroke. Mayo Clin Proc 2008;83:1107–15. 10.4065/83.10.1107 18828970

[14] Keijzer-Veen MG, Euser AM, van et al A regression model with unexplained residuals was preferred in the analysis of the fetal origins of adult diseases hypothesis. J Clin Epidemiol 2005;58:1320–4. 10.1016/j.jclinepi.2005.04.004 16291478

[15] Wills AK, Lawlor DA, Matthews FE, et al Life course trajectories of systolic blood pressure using longitudinal data from eight UK cohorts. PLoS Med 2011;8:e1000440 10.1371/journal.pmed.1000440 21695075 PMC3114857

[16] Arnlöv J, Lind L, Sundström J, et al Insulin resistance, dietary fat intake and blood pressure predict left ventricular diastolic function 20 years later. Nutr Metab Cardiovasc Dis 2005;15:242–9. 10.1016/j.numecd.2004.10.002 16054547

[17] Vasan RS, Benjamin EJ Diastolic heart failure—no time to relax. N Engl J Med 2001;344:56–9. 10.1056/NEJM200101043440111 11136963

[18] Zile MR, Baicu CF, Bonnema DD Diastolic heart failure: definitions and terminology. Prog Cardiovasc Dis 2005;47:307–13. 10.1016/j.pcad.2005.02.006 16003645

[19] Gandhi SK, Powers JC, Nomeir AM, et al The pathogenesis of acute pulmonary edema associated with hypertension. N Engl J Med 2001;344:17–22. 10.1056/NEJM200101043440103 11136955

[20] Beckett NS, Peters R, Fletcher AE, et al Treatment of hypertension in patients 80 years of age or older. N Engl J Med 2008;358:1887–98. 10.1056/NEJMoa0801369 18378519

[21] Ahmed A, Rich MW, Fleg JL, et al Effects of digoxin on morbidity and mortality in diastolic heart failure: the ancillary digitalis investigation group trial. Circulation 2006;114:397–403. 10.1161/CIRCULATIONAHA.106.628347 16864724 PMC2628473

[22] Solomon SD, Janardhanan R, Verma A, et al Effect of angiotensin receptor blockade and antihypertensive drugs on diastolic function in patients with hypertension and diastolic dysfunction: a randomised trial. Lancet 2007;369:2079–87. 10.1016/S0140-6736(07)60980-5 17586303

[23] Tapp RJ, Sharp A, Stanton AV, et al Differential effects of antihypertensive treatment on left ventricular diastolic function: an ASCOT (Anglo-Scandinavian Cardiac Outcomes Trial) substudy. J Am Coll Cardiol 2010;55:1875–81. 10.1016/j.jacc.2009.11.084 20413040

[24] Barron AJ, Hughes AD, Sharp A, et al Long-term antihypertensive treatment fails to improve E/e′ despite regression of left ventricular mass: an Anglo-Scandinavian cardiac outcomes trial substudy. Hypertension 2014;63:252–8. 10.1161/HYPERTENSIONAHA.113.01360 24218432

[25] Shah SJ, Gheorghiade M Heart failure with preserved ejection fraction: treat now by treating comorbidities. JAMA 2008;300:431–3. 10.1001/jama.300.4.431 18647986

[26] Lehmann MV, Zeymer U, Dechend R, et al Ambulatory blood pressure monitoring: is it mandatory for blood pressure control in treated hypertensive patients?: prospective observational study. Int J Cardiol 2013;168:2255–63. 10.1016/j.ijcard.2013.01.209 23474245

[27] Hill MN, Miller NH, Degeest S, et al Adherence and persistence with taking medication to control high blood pressure. J Am Soc Hypertens 2011;5:56–63. 10.1016/j.jash.2011.01.001 21320699

[28] Haider AW, Larson MG, Franklin SS, et al Systolic blood pressure, diastolic blood pressure, and pulse pressure as predictors of risk for congestive heart failure in the Framingham Heart Study. Ann Intern Med 2003;138:10–16. 10.7326/0003-4819-138-1-200301070-00006 12513039

[29] Franklin SS, Levy D Aging, blood pressure, and heart failure: what are the connections? Hypertension 2011;58:760–2. 10.1161/HYPERTENSIONAHA.111.179119 21947472 PMC4711260

[30] Quiñones MA Assessment of diastolic function. Prog Cardiovasc Dis 2005;47:340–55. 10.1016/j.pcad.2005.02.009 16003649

