# Supplementary material for: Midlife blood pressure predicts future diastolic dysfunction independently of blood pressure
Source: Heart. 2016 Apr 7;102(17):1380–7. doi: 10.1136/heartjnl-2015-308836 (PMC4998951; doi:10.1136/heartjnl-2015-308836)
Supplement: Supplementary table — Cardiac risk factor characteristics of study participants [file heartjnl-2015-308836supp_table.pdf]

<u>Online Supplementary Table</u>

Supplementary Table 1. Cardiac risk factor characteristics of study participants

Variable (at age 60-64y unless stated otherwise)	All		Men		Women	
	n	Result	n	Result	n	Result
Haemoglobin A_{1C}, %	1527	5.8 (0.6)	740	5.8 (0.7)	787	5.8 (0.6)
Fasting glucose, mmol/L	1549	5.8 (1.2)	751	6.0 (1.2)	798	5.6 (1.2)
Total cholesterol, mmol/L	1529	5.7 (1.2)	741	5.3 (1.1)	788	6.0 (1.2)
HDL[§§], mmol/L	1529	1.6 (0.4)	741	1.4 (0.3)	788	1.8 (0.4)
Triglycerides, mmol/L [‖‖]	1529	1.3 (0.7)	741	1.4 (0.8)	788	1.2 (0.6)
Diabetes, n (%)	1498	85 (6)	717	43 (6)	781	42 (5)
Smoking habit						
Current, n (%)	144	(10)	74	(10)	70	(9)
Ex, n (%)	621	(41)	350	(49)	271	(35)
Never, n (%)	733	(49)	298	(41)	435	(56)

Data are mean (SD), [‖‖]median (interquartile range) or n (%) as appropriate. Abbreviations: [§§]High density lipoprotein
